# Genomic insights into clinical non-O1/non-O139 *Vibrio cholera*e isolates in Japan

**DOI:** 10.1128/spectrum.00175-25

**Published:** 2025-06-24

**Authors:** Yohei Kobayashi, Masato Suzuki, Shuntaro Umeda, Keisuke Oka, Kenichi Takahashi, Keigo Shibayama, Shinichiro Shibata

**Affiliations:** 1 Department of Microbiology, Nagoya City Public Health Research Institute, Nagoya, Japan; 2 Department of Bacteriology, Nagoya University Graduate School of Medicine, Nagoya, Japan; 3 Antimicrobial Resistance Research Center, National Institute of Infectious Diseases, Tokyo, Japan; 4 Department of Infectious Diseases, Nagoya University Hospital 36590, Nagoya, Japan; Universidad Andres Bello, Santiago, Chile

**Keywords:** non-O1/non-O139 *Vibrio cholerae *(NOVC), whole-genome sequencing, virulence factor gene, T3SS, T6SS

## Abstract

Non-O1/non-O139 *Vibrio cholerae* (NOVC) causes sporadic intestinal infections and systemic symptoms. Recently, global warming has led to an increase in NOVC infections; however, there have been no reports on the genomic analysis of clinical NOVC isolates in Japan, and the molecular understanding of NOVC remains inadequate. We identified three cases of NOVC infection in Nagoya, Japan, in 2020 and performed genomic analysis to reveal the molecular characteristics of these isolates and compared them with those of previously reported clinical cases. Phylogenetic analysis revealed high diversity. This result indicates that the three cases were sporadic within the area over a short period of time from multiple contaminated sources. However, most clinical isolates carried virulence factor genes encoding El Tor type hemolysin, type VI secretion system (T6SS), and repeats-in-toxin toxin, and approximately half of the isolates carried *nanH* encoded on VPI-2, as well as the type III secretion system (T3SS). Additionally, detailed whole-genome sequencing (WGS) analysis revealed various types of T3SS, T6SS, VPI-2, and VSP-2. Variations in the number of Rep1 units of VopM were observed in the T3SS of the NOVC isolates in this study. The T6SS type identified in this study consisted of a large cluster and auxiliary clusters, showing diversity among the NOVC isolates. We recommend continued monitoring of clinical NOVC isolates as horizontal transmission along with host and environmental recombination may lead to changes in virulence.

**IMPORTANCE:**

Although reports of non-O1/non-O139 *Vibrio cholerae* (NOVC) infections are rare, their actual incidence remains uncertain. This is partly due to nonspecific symptoms, the absence of a surveillance system in most countries including Japan, and the lack of appropriate laboratory culture techniques. However, NOVCs in the environment are increasing due to global warming, and the risk of NOVC infections is increasing. In this study, we conducted a comprehensive genomic analysis of clinical NOVC isolates from a city in Japan and compared their virulence factor profiles with those of previously reported clinical isolates using whole-genome sequencing (WGS). This result indicates that some sporadic cases have occurred in the area, suggesting that there are multiple sources of NOVC infection. The accumulation of such data will enhance our understanding of the pathogenicity of NOVCs and improve diagnostic accuracy.

## INTRODUCTION

*Vibrio cholerae* is a pathogenic aerobic gram-negative bacillus that causes waterborne diseases. *V. cholerae* is classified into more than 200 serotypes based on the O-antigen polysaccharides (1). Among the serotypes, clones belonging to serotypes O1 and O139 are the leading causes of cholera epidemics and pandemics owing to their ability to produce cholera toxin (CT). Cholera toxin phage (CTXφ), the CT-encoding bacteriophage, integrates into the genomes of these *V. cholerae* serotypes. The other *V. cholerae* serotypes are referred to as non-O1/non-O139 *V. cholerae* (NOVC). Most NOVC clones are incapable of producing CT and cause sporadic infections such as diarrhea, soft tissue infections, and bacteremia. NOVC infections are primarily associated with the consumption of contaminated seafood products such as oysters, crabs, and shrimp (2).

Although NOVC does not harbor CTXφ, NOVC often possesses several other virulence factors, including hemolysin, repeats-in-toxin (RTX) toxin, hemagglutinin protease (HAP), heat-stable enterotoxin (NAG-ST), cholix toxin, type III secretion system (T3SS), and type VI secretion system (T6SS) (2–8). In addition to CTXφ, *V. cholerae* pathogenicity islands (PAIs), including VPI-1, VPI-2, VSP-1, and VSP-2, have been reported and implicated in bacterial pathogenesis and pathogenicity, as well as cholera pandemic dissemination (9). Although there are numerous reports on virulence factors other than CT, research efforts on *V. cholerae* have been mainly focused on CT-producing serotypes.

In recent years, global warming has led to an increase in contamination rates and infections of *Vibrio* spp., including NOVC (10–12). In Japan, the government has implemented regulations to prevent *Vibrio* spp. foodborne outbreaks, which has led to a decrease in the number of *Vibrio* spp. infections such as food poisoning by *V. parahaemolyticus* (13). In addition, the occurrence of CT-producing *V. cholerae* infection has remained at less than 10 cases in the last decade in Japan. However, recent increases in seawater temperatures around Japan have elevated the risk of NOVC infections, with reports of such cases in Japan (14, 15). NOVC infections are often underdiagnosed due to a lack of experience in identifying *Vibrio* infections and because many diagnostic and clinical laboratories do not use appropriate culture media such as thiosulfate-citrate-bile salts-sucrose (TCBS) agar (11). Additionally, there is no reporting system for NOVC infections in most countries, including Japan, such that an accurate estimation of the number of cases is improbable; the actual number of infections is likely to be even higher. Community-acquired infections in NOVCs are considered common in immunocompromised patients and those with liver disease (16).

In 2020, three patients presented with cholera symptoms, and each was tested for *V. cholerae* infection and finally diagnosed with NOVC infection at hospitals in Nagoya City, Japan. In this study, we aimed to elucidate the molecular characteristics, virulence factor gene profiles, and antimicrobial susceptibility profiles of NOVC isolates and molecular epidemiological comparison of clinical NOVC isolates, including those from previous studies.

## RESULTS

### Bacterial identification and multilocus sequence typing analysis

**Fig 1:**
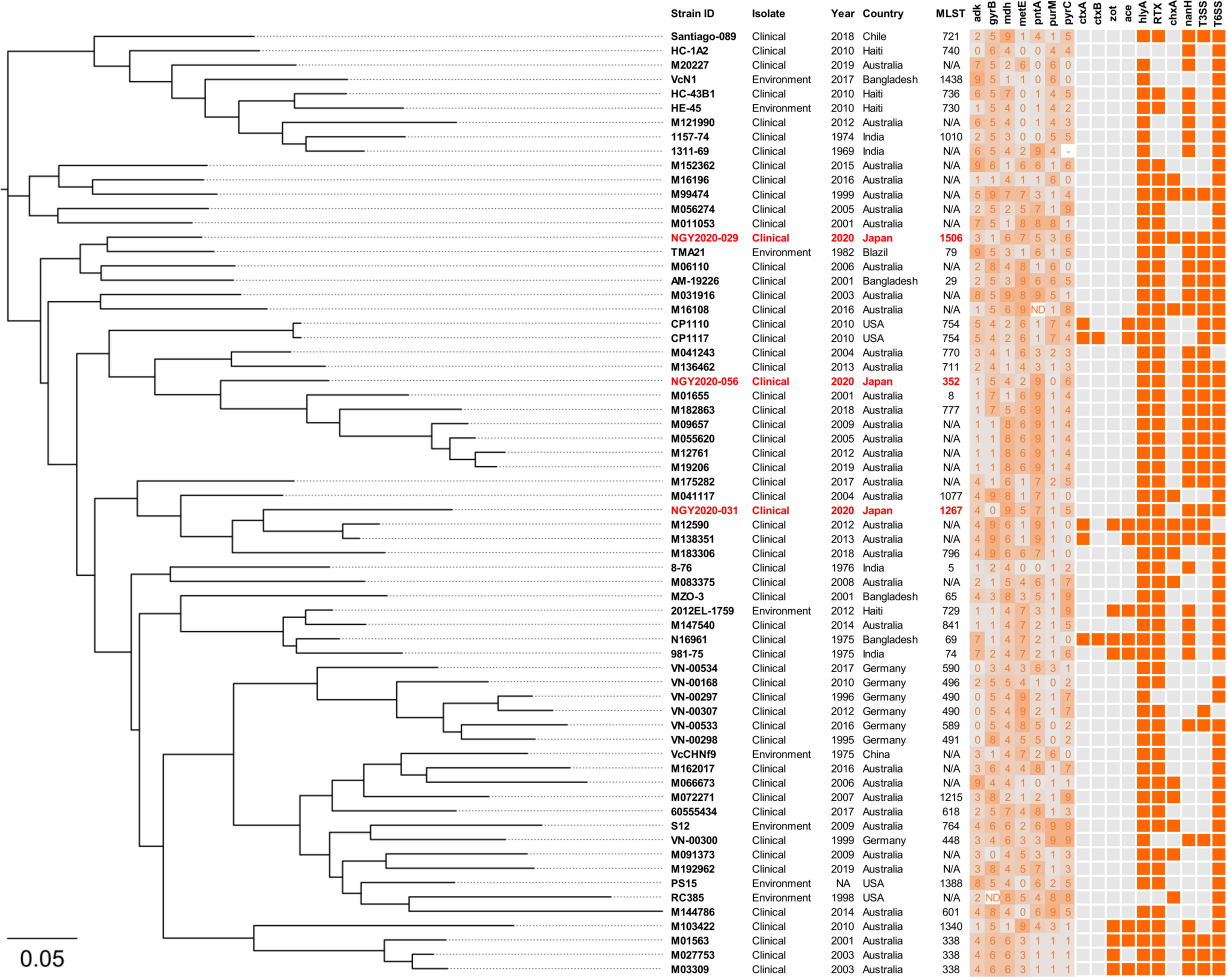
Phylogenetic tree based on pan-genome analysis using whole-genome sequencing data from the present isolates and previously reported clinical isolates of non-O1/non-O139 *Vibrio cholerae*, along with a search for virulence factor genes using the Virulence Factor Database. Phylogenetic and virulence factor gene detection analyses using whole-genome sequencing data of 57 clinical non-O1/non-O139 *Vibrio cholerae* isolates, including the isolates in this study and seven environmental strains as references in the public database, revealed genetic lineage diversity. Among the clinical isolates, 98.2% (56/57) harbored *hlyA*, 93.0% (53/57) harbored the type IV secretion system, 86.0% (49/57) harbored repeats-in-toxin toxin clusters, 61.4% (35/57) harbored *nanH*, and 47.4% (27/57) harbored the type III secretion system. The cholera toxin gene was detected in 7.0% (4/57) of the isolates.

Two *V. cholerae* isolates, NGY2020-031 and NGY2020-056, were obtained from patients’ feces, and one isolate, NGY2020-029, was obtained from the patient’s blood cultures (Table S1). These isolates were confirmed as *V. cholerae* based on typical biochemical characteristics and were further identified as NOVC using PCR. Multilocus sequence typing (MLST) analysis revealed that the isolates had different sequence types: ST352, ST1267, and ST1506 (Fig. 1). All seven loci had different allele numbers, indicating that NOVC cases were sporadic. A PubMLST database search revealed that ST352 has been reported in Burkina Faso, ST1267 has been reported in China, and ST1506 has never been reported.

### Antimicrobial susceptibility test

Antimicrobial susceptibility testing revealed that all isolates were susceptible to ampicillin, azithromycin, chloramphenicol, tetracycline, and sulfamethoxazole-trimethoprim, which are the primary agents outlined by the Clinical and Laboratory Standards Institute (CLSI). In addition, none of the isolates were resistant to cefazolin, cefotaxime, cefepime, imipenem, meropenem, or levofloxacin (Table S2). The WGS-based screening of antimicrobial resistance (AMR) genes using ResFinder did not detect any known AMR genes.

### Virulence factor gene profile of NOVC in this study

In the virulence factor gene screening, CT genes—*ctxA* and *ctxB*, zonula occludens toxin gene—*zot*, accessory cholera enterotoxin gene—*ace*, and toxin-controlled pili gene—*tcp*, which are related to CTXφ coding, were not detected in all isolates in this study. Among the other virulence factor genes tested, the NAG-ST gene—*snt*—was not detected, whereas the El Tor variant hemolysin gene—*hly-ET*; HAP gene—*hap*; RTX toxin genes—*rtxA*, *rtxB*, *rtxC*, and *rtxD*; and neuraminidase encoding gene—*nanH*—were detected in all isolates in this study. *chxA*, which encodes the cholix toxin, was detected only in NGY2020-031, and its subtype was *chxAI* (Table S3).

### Detailed analysis of T3SS and T6SS in NOVC from this study

**Fig 2:**
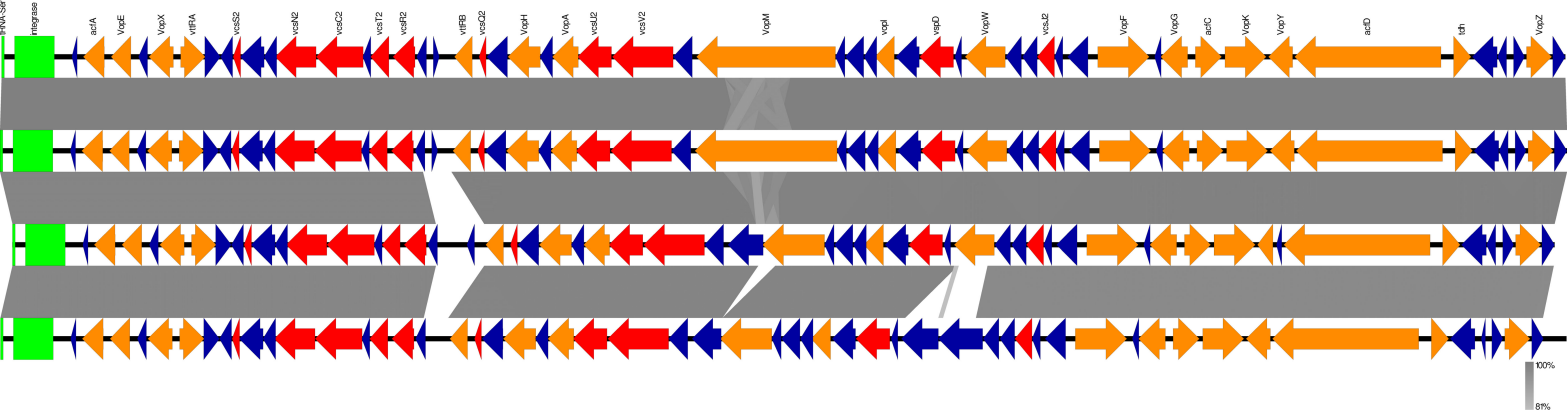
Comparison of type III secretion system (T3SS) genes detected in non-O1/non-O139 *Vibrio cholerae* isolates through whole-genome sequencing reveals predominantly conserved T3SS clusters in clinical isolates. T3SS gene cluster regions were compared using Easyfig. The figures are shown in the order of AM-19226, such as the reference non-O1/non-O139, and *Vibrio cholerae* strains harboring T3SS, NGY2020-029, NGY2020-031, and NGY2020-056 from the top to the bottom. Red, orange, and dark blue arrows indicate T3SS component genes, identified genes, and hypothetical proteins, respectively. T3SS membrane-associated pore-forming complex genes and ATPases required for secretion, T3SS-associated translocator genes, transcriptional regulator genes, and virulence factor genes were detected in all isolates, although VopM and VopW differed among these isolates.

Initial PCR screening for virulence factor genes detected T3SS structural component genes such as *vcsC*, *vcsV, vcsN*, and *vspD* in all isolates. Because the T3SS contains effector proteins associated with virulence, detailed sequence comparisons were performed using the WGS data. The T3SS gene clusters of the isolates in this study were compared to the genomic data of *V. cholerae* AM-19226, a reference strain possessing the T3SS gene cluster. All isolates contained T3SS membrane-associated pore-forming complex genes such as *vcsV2*, *vcsU2*, *vcsR2*, *vcsT2*, *vcsS2*, *vcsC2*, and *vcsQ2*; an ATPase required for secretion such as *vcsN2*; a T3SS-associated translocator gene such as *vspD*; transcriptional regulator genes such as *vttR*_A_ and *vttR*_B_; virulence factor genes such as *acfA*, *acfC*, and *acfD*; and effector protein genes such as *vopE*, *vopX*, *vopH*, *vopA*, *vopF*, *vopG*, *vopK*, *vopY*, and *vopZ* (Fig. 2). Comparative analysis confirmed that the VopM regions differed in size; thus, amino acid sequence comparisons were performed: VopM of NGY2020-029 consisted of 1,464 amino acids, VopM of NGY2020-031 consisted of 638 amino acids, VopM of NGY2020-056 consisted of 532 amino acids, and VopM of AM-19226 consisted of 1,432 amino acids. We performed repeat region analysis and detected an Rep1-like region of VopM with seven repeats in NGY2020-029, three repeats in NGY2020-031, and two repeats in NGY2020-056 (similarity: 17.1–100.0%; Fig. S1). The fourth Rep1-like region of VopM in NGY2020-029 has the same sequence as the fifth Rep1-like region of VopM in NGY2020-029 and the fourth Rep1-like region of VopM in AM-19226. Amino acid mutations were detected in the VopW of NGY2020-056, and the similarity between the VopW of AM-19226 and that of NGY2020-056 was 62.1%.

Screening of the T6SS region revealed that all isolates harbored multiple T6SS regions. The presence of T6SS diversity of *V. chorelae* has been reported, and we performed T6SS typing using WGS data to assess this diversity. T6SS analysis detected a large cluster and two auxiliary clusters, AUX-1 and AUX-2, in all isolates (Fig. S2). In the large cluster, the effector proteins of the three isolates were found to be diverse, such as types C, E, and complex types, including types A, C, and E, although the effector proteins of all isolates were identified as type C effector proteins. In AUX-1, all isolates contained type C effector proteins and immunoproteins, whereas only NGY2020-029 had an actin cross-linking domain (ACD) at the tip of the effector protein. In AUX-2, NGY2020-029 was type E, NGY2020-031 was type A, and NGY2020-056 was type D. AUX-3 was not detected in any isolate in this study. Other T6SS regions were identified in the database search: AUX-4, which has a sequence similar to that of the T6SS of the genomic island GI*Vch*S12, was detected in NGY2020-031, and type C AUX-5 was detected in NGY2020-056, as opposed to previous sequences (17).

### Pan-genome analysis with the virulence profile in clinical NOVC isolates

We performed phylogenetic analysis to assess the cluster of clinical isolates using previous genomic data from clinical isolates. Phylogenetic and gene detection analyses using WGS data from 58 NOVC clinical and seven environmental isolates as reference strains in the public database revealed genetic lineage diversity (Fig. 1). For instance, no representative ST types related to different countries have been identified, and a phylogenetic tree with several ST types was widely distributed. The three isolates in this study also clustered in phylogenetically distinct clades, and no relationship was observed between them. Among the clinical isolates, 98.2% (56/57) harbored *hlyA*, 93.0% (53/57) harbored T6SS, 86.0% (49/57) harbored RTX toxin clusters, 61.4% (35/57) harbored *nanH*, and 47.4% (27/57) harbored T3SS (Fig. 1). CTX was detected in 7.0% (4/57) of the isolates.

### Genome comparison of PAI regions in NOVC isolates

**Fig 3:**
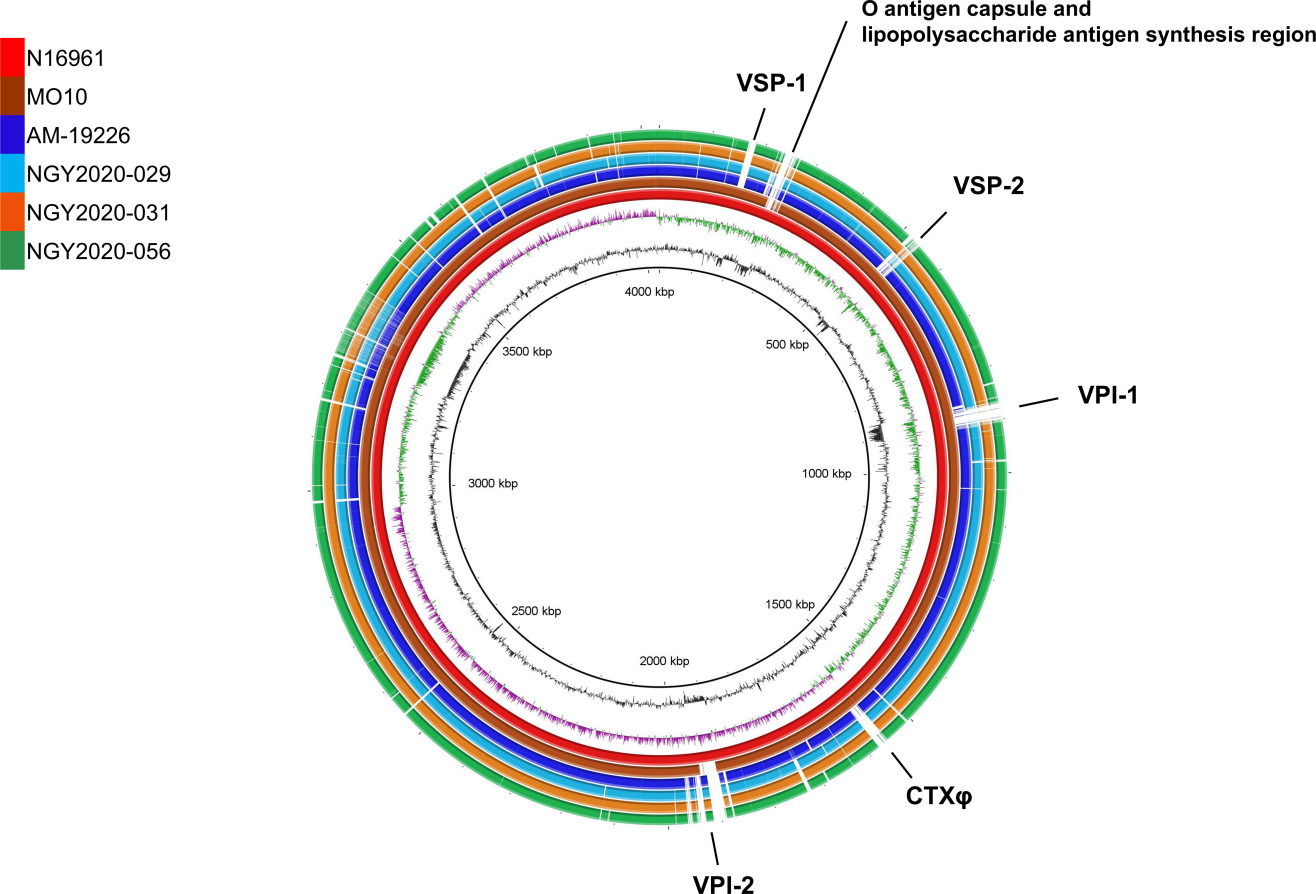
Genome map comparison of non-O1/non-O139 *Vibrio cholerae* isolates using the BLAST Ring Image Generator (BRIG) tool. Data comparisons for each draft genome were performed using BRIG. The reference data used to create the genome map were drawn using the N16961 strain, which is an El Tor type O1 *Vibrio cholerae* strain. N16961 is shown in red; MO10, the O139 *V. cholerae* strain, in brown; AM-19226, the O1 and O139 *V. cholerae* strains in blue; NGY2020-029 in sky blue; NGY2020-031 in orange; and NGY2020-056 in green. All strains in this study were found to be deficient in the cholera toxin phage, VPI-1, and VSP-1. In the VPI-2 region, all isolates in this study had a gene distribution similar to that of AM-19226, whereas NGY2020-031 harbored a fragmented VSP-2 region.

A BLAST Ring Image Generator (BRIG) analysis was performed to assess the presence of PAIs and genomic similarities in each isolate. CTXφ, VPI-1, and VSP-1 were absent in all isolates, while VPI-2 was fragmented in all isolates and VSP-2 was conserved in NGY2020-031 (Fig. 3). To assess the detailed genetic possession of PAIs in the isolates, PAIs were identified using the VicPred database and compared with each N16961 locus (Table 1). Among VC1758 to VC1809 in the VPI-2 region, VC1758, VC1773–1779, VC1781–1787, and VC1804 were detected in all isolates. In contrast, VC1759 was only detected in NGY2020-031 and NGY2020-056 and VC1805–1809 in NGY2020-029 and NGY2020-031. All strains possessed the VC1784 region encoding the neuraminidase gene *nanH*, which is involved in CT modification. VSP-2 regions NGY2020-029 and NGY2020-056 were found to be lacking VC490–VC500 and VC502–VC516, respectively. NGY2020-031 harbored VC490–498, VC504–510, and VC516, which are similar to the TMA21 strain isolated from the environment in Brazil among typical VSP-2 structures, but with the deletion of VC0501a–VC0501b (18).

**TABLE 1:** Diversity of pathogenicity island distribution loci, including VPI-2 and VSP-2, in non-O1/non-O139 *Vibrio cholerae* isolates in this study

	VPI-2										VSP-2									
	VC1758	VC1759	VC1760 VC1772	VC1773 VC1779	VC1780	VC1781 VC1784	VC1785	VC1786 VC1787	VC1788 VC1803	VC1804	VC1805 VC1809	VC0489	VC0490 VC0498	VC0499 VC0500	VC0501a VC0501b	VC0502 VC0503	VC0504 VC0510	VC0511 VC0515	VC0516	VC0517
NGY2020-029	■^*a*^	-^*b*^	-	■	-	■	■	■	-	■	■	■	-	-	■	-	-	-	-	■
NGY2020-031	■	■	-	■	-	■	■	■	-	■	■	■	■	-	-	-	■	-	■	■
NGY2020-056	■	■	-	■	-	■	■	■	-	■	-	■	-	-	■	-	-	-	-	■

^*a*^ “■” indicates a positive result as determined by Vicpred.

^*b*^ “–” indicates a negative result as determined by Vicpred.

## DISCUSSION

Three cases of NOVC infection were identified in Nagoya City, Japan, in 2020. Using MLST analysis, we demonstrated that all the isolates had distinct genetic backgrounds, indicating that the infections were sporadic and caused by different contaminated sources. In previous studies, the antimicrobial susceptibility of NOVCs tended to vary among regions; however, all isolates in this study were broadly susceptible to antimicrobial agents, suggesting that these isolates derived from areas with low AMR bacterial contamination (19–21). Pan-genome analysis supported the MLST results and demonstrated the diversity of clinical NOVC isolates. The resolution of MLST analysis between strains is adequate, and it is a rapid and useful method for detecting common NOVC contamination (4, 8).

Assessment of virulence factor genes in clinical NOVC strains showed that El Tor type hemolysin, T6SS, and RTX toxin were the major virulence factors, and approximately half of the isolates that harbored *nanH* encoded VPI-2 and T3SS. The high prevalence of El Tor *hlyA*, T6SS, and RTX toxins in environmental strains may also be responsible for their high detection rates in clinical isolates (6, 8, 20, 22, 23). The isolates exhibit hemolytic activity due to the El Tor type hemolysin and utilize the T6SS to maintain their dominance in the environment and *in vivo* and are considered pathogenic (24–26). The advantage of WGS is its ability to comprehensively detect virulence factor genes, not only *rtxA* and *rtxC* but also *rtxABCD* as an RTX toxin cluster (27). Full-length RTX toxin clusters including *rtx*ABCD, one of the most important factors associated with clinical cases, were harbored in 86.0% clinical strains. All isolates in this study harbored full-length RTX toxin clusters, suggesting their ability to secrete RTX toxin. This toxin induces clinical symptoms such as diarrhea through actin cytoskeletal disruption via ACD and Rho GTPase inactivation domains and promotes colonization of the small intestine by suppressing inflammation (28).

Despite the low prevalence of *nanH* and T3SS in environmental NOVC isolates, approximately half of the clinical isolates in this study carried these genes (6, 8, 20, 22, 23, 29). The sialic acid metabolism region harbors the neuraminidase gene, *nanH*, which is associated with sialic acid catabolism of the GM1 receptor, leading to severe symptoms of cholera and host adaptation (30, 31). As 61.4% of clinical isolates harbor *nanH* encoded in VPI-2, this virulence factor and the VPI-2 region also play important roles in the clinical features of NOVC infection. Previous studies have reported that the T3SS of *V. cholerae* is similar to that of *V. parahaemolyticus* and that T3SS components and effector proteins are associated with clinical symptoms such as diarrhea (5, 7, 19, 32–35). In this study, the T3SS cluster was present as a genomic island in front of the nan-nag region, forming a cluster that contained precise T3SS component genes and pathogenic effector proteins such as VopE, VopF, VopK, VopM, VopW, VopX, and VopY. Repeated mutations in VopM and substitutions in VopW were detected in each strain. VopM is a homolog of VopV in *V. parahaemolyticus* encoding multiple repeats of the Rep1 unit that exhibit F-actin-binding activity. The varying number of Rep1 units in each isolate in this study indicates differences in enterotoxicity, and consequently, diarrheagenic potential. These results suggest that the diversity of T3SS clusters in NOVC depends on T3SS-positive strains and their effector proteins (36, 37).

Comprehensive analysis of phylogenetic relationships among NOVC infections, including genotypes and virulence factor genes, using genomic information from clinical isolates revealed that several virulence factor genes are involved in clinical cases, although no phylogenetic relationships were observed among clinical isolates.

*V. cholera* infections have caused pandemics and outbreaks due to the evolution of specific genotypes such as the El Tor type 7th pandemic strain that has been acquired via horizontal transmission including PAIs such as CTXφ, VPI, and VSP (9). The results of the sequence-based virulence factor gene screening among the isolates showed the same profile, indicating that the isolates were almost all NOVC isolates of the same genetically evolved lineage, and the BRIG images supported this result (Fig. 3). In contrast, the analysis of gene clusters, such as T3SS, T6SS, VPI, and VSP, allowed us to confirm the differences among the isolates. The T6SS genes in *V. cholerae* are organized into large gene clusters that contain structural, regulatory, and toxin components and into auxiliary clusters that also encode structural and toxin elements. To date, two effector types (A and C) have been identified in AUX-1, five types (A–E) in AUX-2, and 12 types (A–G and I–M) in large clusters encoding effector and immunity proteins. Although environmental strains exhibit diverse effector combinations (AUX-1 C/AUX-2 D/Large E, or CDE), pandemic strains, such as El Tor O1 and O139, consistently encode the AAA class of effector proteins (38). The T6SS analysis revealed that the Large Cluster and AUX classes of all isolates were non-AAA classes, such as the CEC of NGY2020-029, CAC of NGY2020-031, and CDC of NGY2020-056, indicating that all were environmental isolates (Fig. S2). We detected multiple immune proteins in the Large Cluster NGY2020-056, suggesting that these isolates acquired diverse immunity protein genes through horizontal transmission. The presence of ACD in AUX-1 of NGY2020-029 may contribute to T6SS-dependent host cell cytotoxicity and impaired phagocytosis (39). Overall, the differences in effector proteins in the T3SS, immune proteins in the T6SS, and VPI and VSP variations indicate that each isolate evolved slightly in the host and environment. Such diversity should lead to increased virulence and creation of novel virulence genotypes; therefore, sequence-based monitoring of NOVC is needed.

This study has some limitations. First, a small sample size was used. Additionally, only genome-based virulence analyses were performed. However, NOVC infection is not included in Japanese surveillance, and the occurrence of NOVC infection is infrequent. Therefore, we believe that the expansion of such genomic information would be beneficial.

### Conclusion

Multiple cases of NOVC infection were identified in the same city in Japan in 2020. MLST and pan-genome analyses revealed no association between the isolates in this study, suggesting that these cases were sporadic and indicating the presence of multiple potential risk factors for NOVC infection, with no dominant virulent strain responsible. Virulence factor gene analysis indicated that El Tor type hemolysin, T6SS, RTX toxin, *nanH*, and T3SS were important risk factors for NOVC infection. Although the virulence factor gene profiles of the isolates in this study were similar, we found that variations in the T3SS, T6SS, VPI-2, and VSP-2 might be related to disease severity. Additionally, these results support the notion that each isolate evolved slightly in response to the host and the environment. As global warming increases the prevalence of NOVC, the potential for such diversity will lead to further increased pathogenicity and the emergence of novel pathogenic genotypes, highlighting the need for sequence-based monitoring of NOVC infections.

## MATERIALS AND METHODS

### Bacterial collection and characterization

In 2020, three bacterial isolates from different hospitals were submitted to Nagoya City Public Health Research Institute for *V. cholerae* testing. To identify *V. cholerae*, the samples were inoculated onto TCBS agar (Eiken Chemical, Tokyo, Japan) and CHROMagar Vibrio agar (Kanto Chemical, Tokyo, Japan), and incubated at 37°C for 24 h. To determine the biochemical characteristics of the isolated strains, they were inoculated onto 2% NaCl-supplemented triple sugar iron agar and 2% NaCl-supplemented lysine indole motility medium, and a salt tolerance test was performed. Bacterial DNA was extracted using the QIAmp DNA Mini Kit (QIAGEN, Hilden, Germany) according to the manufacturer’s protocol. PCR was performed to detect the housekeeping genes of *V. cholerae*, the CT gene, and the O1 and O139 serotypes, as previously described (40, 41).

### Antimicrobial susceptibility testing

Antimicrobial susceptibility testing was performed according to CLSI documentation (42). Ampicillin, cefazolin, cefotaxime, cefepime, imipenem, meropenem, sulfamethoxazole-trimethoprim, and levofloxacin susceptibilities were tested using the microdilution method. In addition, the susceptibility to chloramphenicol and tetracycline was tested using the disk diffusion method with a KB disk (Eiken Chemical), and azithromycin susceptibility was tested using an E-test strip (Biomérieux, Tokyo, Japan).

### Virulence factor gene screening and MLST

Virulence factor gene profiling was performed using PCR, as previously described (Table S4) (20). We determined the presence of the virulence factor genes*—ctxA*, *ctxB*, *ace*, and *zot*—encoded in CTXφ. In addition, we determined the presence of *tcpI*, *tcpA*, and *tcpH-tcpA*, which are associated with CTXφ tolerance and encode a type IV pilus. The presence of other virulence factor genes, including hemolysin, heat-stable enterotoxin (NAG-ST), HAP, RTX toxin, and *nanH*, as well as *vcsC, vcsV, vcsN*, and *vspD* in T3SS and *vasA, vasH*, and *vasK* in T6SS, was also determined. PCR was performed using TaKaRa Ex Taq HS (TaKaRa Bio, Shiga, Japan) according to the manufacturer’s instructions. MLST analysis was performed for Non-O1/non-O139 *V. cholerae* using the PubMLST protocol (6). The nucleotide sequence was assembled using ATGC software (Genetyx, Tokyo, Japan) and submitted to PubMLST to determine the allele number, sequence type, and clonal complex. If a novel allele or ST was identified, it was registered in the PubMLST database.

### Whole-genome sequencing

Genome analysis was performed according to previous reports using the Galaxy Server, and the genome assembly parameters were set according to the previous method (43). Draft genomes were obtained from bacterial DNA using the MiSeq system (Illumina, San Diego, CA, USA). Genomic DNA libraries were prepared using a Nextera XT DNA Library Preparation Kit (Illumina, USA) according to the manufacturer’s protocol. Paired-end sequencing (150 bp) was performed on a MiSeq system using a MiSeq Reagent Kit v2 (Illumina, USA) according to the manufacturer’s protocol. Raw sequence data were trimmed using Trimmomatic v0.38 and *de novo-*assembled into contigs using Shovill v1.1.0 (https://github.com/tseemann/shovill) with SPAdes v3.14.1 (44). AMR genes were detected in the resulting draft genome using ResFinder, which included staramr v0.9.1 (45, 46).

Bacterial species identification using the draft genome and functional annotation of the predicted genes was performed using the DDBJ Fast Annotation and Submission Tool (DFAST) (Table S5) (47). In addition, the assembled genome sequence was assessed for completeness and contamination using DFAST_QC (48). The assembled sequences were submitted to the Virulence Factor Database (VFDB) to identify *V. cholerae* virulence factor genes (49). Additionally, the bioinformatics tool Cholera Finder v1.0, described on the Center for Genomic Epidemiology website (https://cge.food.dtu.dk/services/CholeraeFinder/), was used to screen for virulence factor genes. A phylogenetic tree, based on the pan-genome analysis of both the core and accessory genomes based on single-nucleotide variants within core genomes, was constructed from the WGS data of publicly genome-available isolates of NOVC using Prokka v1.14.6 with default parameters, Roary v3.13.0, and FigTree v1.4.4 (http://tree.bio.ed.ac.uk/software/figtree/) (50, 51). Publicly available NOVC genomic data were obtained from a previous clinical report (Table S5) (3, 8, 29). The phylogenetic tree created is shown in parallel with the pathogenic gene profiles obtained from the VFDB analysis. The constructed contigs were sorted using the Mauve alignment tool with N16961, a *V. cholerae* O1 El Tor type strain, as a reference (52, 53). The T3SS cluster was visualized by Easyfig v2.2.5 with default parameters using the previously reported gene sequence with the AM-19226 strain and *V. cholerae* O39 serogroup as the NOVC reference strains, and each gene was compared (54). T6SS analysis was performed using VFDB to search for T6SS components, and the T6SS Prediction Tool was used to detect the localization of the T6SS cassette in the genome (49, 55). Large clusters, AUX-1, AUX-2, and AUX-5, which are common T6SSs in *V. cholerae*, were subtyped for differences in effector proteins and immune proteins of the T6SS according to previous reports (56–59). We performed an ACD search for the effector proteins of the T6SS using CD-search in NCBI (60). Other T6SS cassettes were characterized through DFAST annotation and a BLAST search using the default database (nr/nt) to characterize the cassettes and compare them with previous reports using SnapGene v7.1.2 (https://www.snapgene.com/) and GENETYX v15 (https://www.genetyx.co.jp/) (47, 61). We conducted a phylogenetic analysis using Roary and compared T6SS possession with that of previously reported representative strains. To identify PAI and genomic differences, the draft genome was visualized using the BRIG tool v0.95 with default parameters (62). The assembled sequences were submitted to Vicpred to search for detailed gene profiles of the VPI and VSP regions (63).

## Data Availability

Draft genome sequences of *V. cholerae* NGY2020-029, NGY2020-031, and NGY2020-056 have been deposited in DDBJ/ENA/GenBank under the accession nos. BAABMQ000000000, BAABUH000000000, and BAABUI000000000, respectively. The raw sequence data are available in the Sequence Read Archive under the accession nos. DRR658979, DRR658980, and DRR658981, respectively.

## SUPPLEMENTAL MATERIAL

Fig. S1 spectrum.00175-25-s0001.TIF Diagram of VopM, including the repeating Rep1 unit, and amino acid sequences of the VopM region detailing the Rep1 unit.

Fig. S2 spectrum.00175-25-s0002.TIF Non-O1/non-O139 *Vibrio cholerae* isolated in this study possess various types of type VI secretion system.

Supplemental tables spectrum.00175-25-s0003.pdf Tables S1 to S5.

Supplemental material spectrum.00175-25-s0004.docx Fig. S1 legend.

Supplemental material 2 spectrum.00175-25-s0005.docx Fig. S2 legend.

ASM does not own the copyrights to Supplemental Material that may be linked to, or accessed through, an article. The authors have granted ASM a non-exclusive, world-wide license to publish the Supplemental Material files. Please contact the corresponding author directly for reuse.
