# Supplementary figures and images for: Genomic insights into clinical non-O1/non-O139 Vibrio cholerae isolates in Japan
Source: Microbiol Spectr. 2025 Jun 24;13(8):e00175-25. doi: 10.1128/spectrum.00175-25 (PMC12323619; doi:10.1128/spectrum.00175-25)

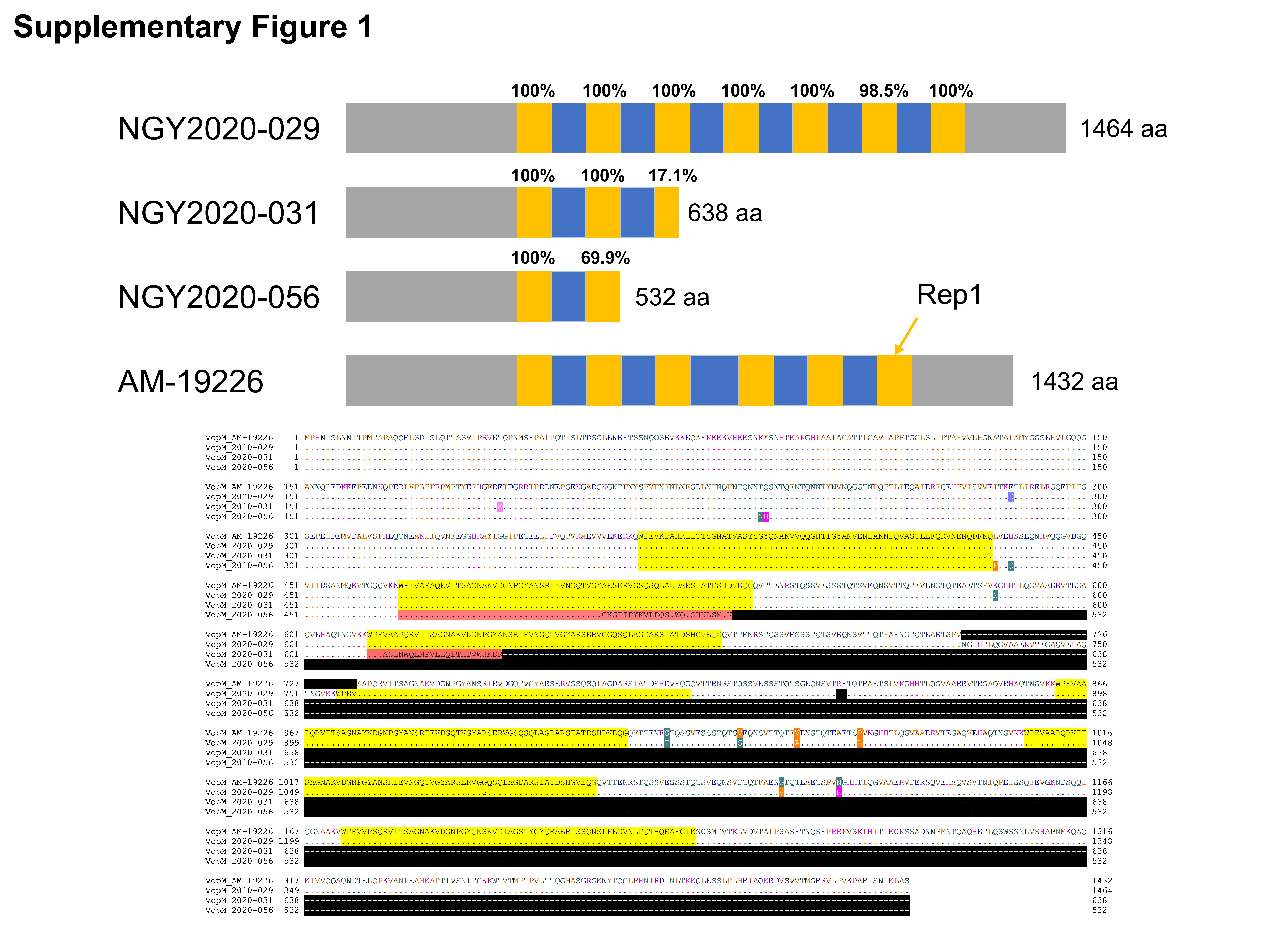

Supplement: Fig. S1 — Diagram of VopM, including the repeating Rep1 unit, and amino acid sequences of the VopM region detailing the Rep1 unit. [file spectrum.00175-25-s0001.tif]

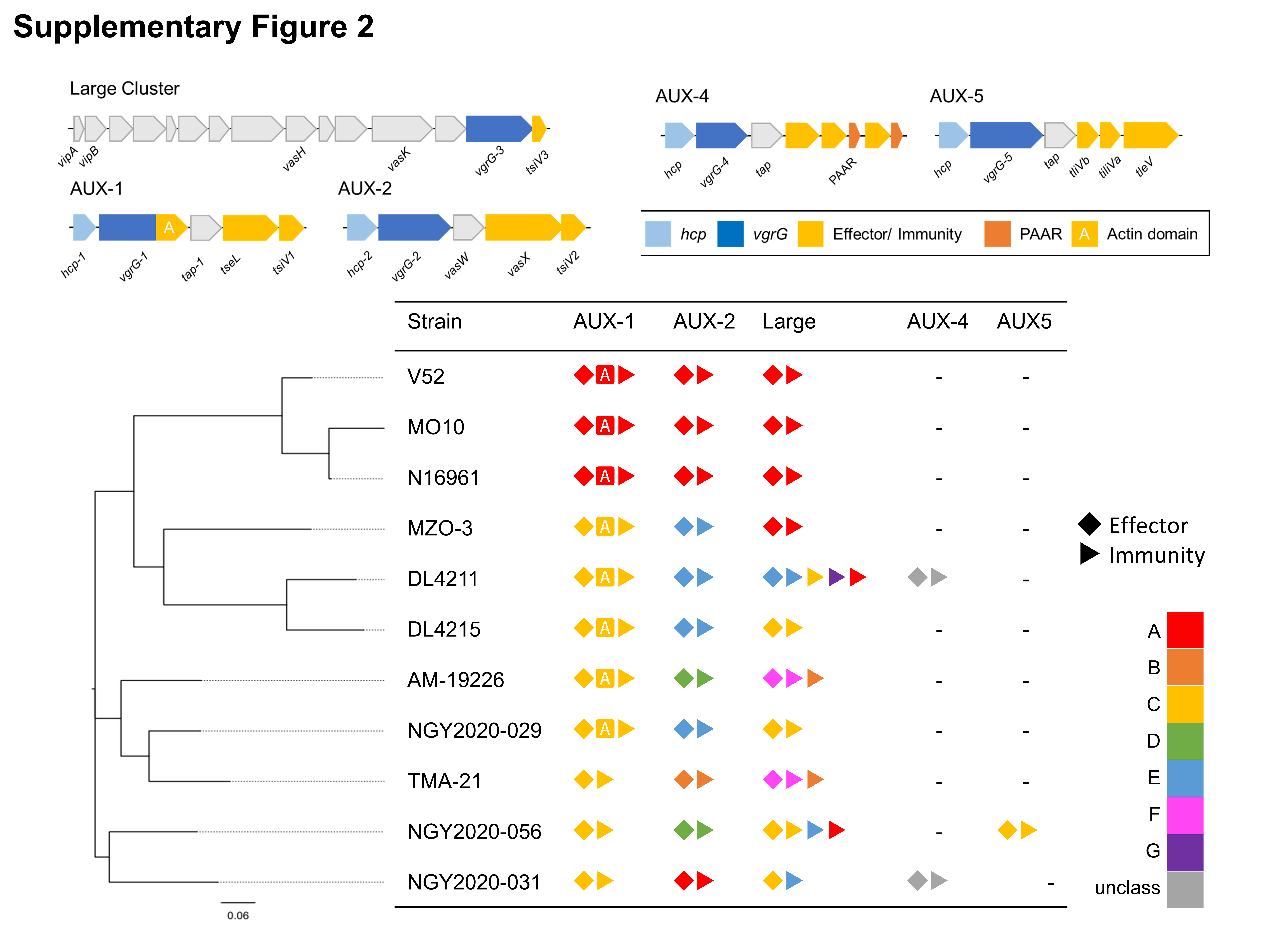

Supplement: Fig. S2 — Non-O1/non-O139 Vibrio cholerae isolated in this study possess various types of type VI secretion system. [file spectrum.00175-25-s0002.tif]
