# Supplementary material for: Genomic insights into clinical non-O1/non-O139 Vibrio cholerae isolates in Japan
Source: Microbiol Spectr. 2025 Jun 24;13(8):e00175-25. doi: 10.1128/spectrum.00175-25 (PMC12323619; doi:10.1128/spectrum.00175-25)
Supplement: Supplemental tables — Tables S1 to S5. [file spectrum.00175-25-s0003.pdf]

Table S1. Genomic information on clinical non-O1/non-O139 *Vibrio cholerae* isolates in Japan in this study

Isolate	Year	Country	Specimen	MLST	Genome Size (bp)	Contig	Coverage	N50	Completeness	Contamination	Accesion no.
NGY2020-029	2020	Japan	Blood	ST1506	3,977,089	102	98x	630,086	99.54	1.54	BAABMQ000000000
NGY2020-031	2020	Japan	Feces	ST1267	4,083,523	65	100x	443,583	99.69	1.46	BAABUH000000000
NGY2020-056	2020	Japan	Feces	ST352	3,923,593	58	95x	246,594	99.46	0.88	BAABUI000000000

Table S2, Results of antimicrobial susceptibility testing of non-O1/non-O139 *Vibrio cholerae* isolates in this study

	AMP MIC (μg/mL)	AZM MIC (μg/mL)	CHL Zone Diameter(mm)	TET Zone Diameter(mm)	SXT MIC (μg/mL)	CFZ MIC (μg/mL)	CRO MIC (μg/mL)	FEP MIC (μg/mL)	IPM MIC (μg/mL)	MEM MIC (μg/mL)	LVX MIC (μg/mL)
Method	Broth Microdilution	E-test	Disk diffusion (30 μg)	Disk diffusion (30 μg)	Broth Microdilution	Broth Microdilution	Broth Microdilution	Broth Microdilution	Broth Microdilution	Broth Microdilution	Broth Microdilution
NGY2020-029	<2	0.125	32	25	<2/38	4	<1	<2	0.5	<0.25	<0.12
NGY2020-031	<2	0.125	32	25	<2/38	2	<1	<2	0.5	<0.25	<0.12
NGY2020-056	<2	0.125	32	25	<2/38	4	<1	<2	0.5	<0.25	<0.12

Table S3. Results of PCR-based virulence factor gene screening in non-O1/non-O139 *Vibrio cholerae* isolated in this study.

		NGY2020-029	NGY2020-031	NGY2020-056
MLST	ST	1506	1267	352
CTXφ	*ctxA*	-	-	-
	ctxB	-	-	-
	zot	-	-	-
	ace	-	-	-
TCP	*tcpAa*	-	-	-
	tcpI	-	-	-
	tcpH - tcpA	-	-	-
Other Toxin	*hlyA*	El Tor type	El Tor type	El Tor type
	stn	-	-	-
	rtxA	+	+	+
	hap	+	+	+
	nanH	+	+	+
TTSS	*vcsC*	+	+	+
	vspD	+	+	+
	vcsN	+	+	+
	vcsV	+	+	+
T6SS	*vasA*	+	+	+
	vasK	+	+	+
	vasH	+	+	+

Table S4. PCR primers used in this study and their target gene information

Target	Gene	Primer	Sequence (5'-3')	Annealin temparatue (°C)	Amplicon size (bp)
CTXφ related genes	*ctxA*	VCT1	ACAGAGTGAGTACTTTGACC	58	308
		VCT2	ATACCATCCATATATTTGGGAG		
	ctxB	ctxB-F	ATGCACATGGAACACCTCAAAATATTACTG	60	231
		ctxB-R	TCCTCAGGGTATCCTTCATCCTTTCAATC		
	zot	225-F	TCGCTTAACGATGGCGCGTTTT	60	947
		1129-R	AACCCCGTTTCACTTCTACCCA		
	ace	Ace-F	TGATGGCTTTACGTGGCTTGTGATC	60	134
		Ace-R	GCCTGTTGGATAAGCGGATAGATGG		
CTXφ tolerance and type IV pilus-encoding genes	*tcpI*	132-F	TAGCCTTAGTTCTCAGCAGGCA	60	862
		951-R	GGCAATAGTGTCGAGCTCGTTA		
	tcpA	72-F	CACGATAAGAAAACCGGTCAAGAG	60	481 (El Tor)
		477-R	CGAAAGCACCTTCTTTCACGTTG		620 (classical)
		647-R	TTACCAAATGCAACGCCGAATG		
		tcA-F	ATGCAATTATTAAAACAGCTTTTTAAG	59	627 (atypical)
		tcA-R	TTAGCTGTTACCAAATGCAACAG		
	tcpH-tcpA	tcpH-1	AGCCGCCTAGATAGTCTGTG	52	1,289
		tcpA-4	TCGCCTCCAATAATCCGAC		
Other virulence factor genes	*hlyA*	489-F	GGCAAACAGCGAAACAAATACC	60	481 (El Tor)
		744-F	GAGCCGGCATTCATCTGAAT		738/727 (classical)
		1184-R	CTCAGCGGGCTAATACGGTTTA		
	stn-sto	67-F	TCGCATTTAGCCAAACAGTAGAAA	55	172
		194-R	GCTGGATTGCAACATATTTCGC		
	hap	Hap-F	ACGTTAGTGCCCATGAGGTC	60	351
		Hap-R	ACGGCAAACACTTCAAAACC		
	rtxA	Rtx-F	CTGAATATGAGTGGGTGACTTACG	60	417
		Rtx-R	GTGTATTGTTCGATATCCGCTACG		
	nanH	nanH-F	CTTCCTCCAATACGGTTCTTGTCTCTTATGC	60	314
		nanH-R	TTCGGCTACCATCGGCAACTTGTATC		
Type III secretion system-encoding genes	*vcsC*	vcsC2-F	GGAAAGATCTATGCGTCGACGTTACCGATGCTATGGG	60	535
		vcsC2-R	CATATGGAATTCCCGGGATCCATGCTCTAGAAGTCGGTTGTTTCGGTAA		
	vcsV	vcsV2-F	ATGCAGATCTTTTGGCTCACTTGATGG	60	742
		vcsV2-R	ATGCGTCGACGCCACATCATTGCTTGC		
	vcsN	vcsN2-F	GGATCCCGGGAATTCCATATGCGTCGACAGTTGAGCCAATTCCATT	60	484
		vcsN2-R	CGGGGTACCATGCTCTAGACGACCAAACGAGATAAT		
	vspD	vspD-F	ATCGTCTAGAACTCGAAGAGCAGAAAAAAGC	60	422
		vspD-R	ATCGGTCGACCTTCCCGCTTTTGATGAAAT		
Type IV Secretion System-encoding genes	*vasH*	vasH-857F	GTGGCACGCTATTTCTGGAT	60	385
		vasH-1242R	TTTCAGCTCACGCACATTTC		
	vasA	vasA-104F	GTACGACCGATCCTGACGTT	60	342
		vasA-446R	ATCTGAATGGTCGTGGCTTC		
	vasK	vasK-1851F	GCGTCAAATTCAGGAAGAGC	60	399
		vasK-2250R	CTGTCCCAGAACCCAACTGT		

Table S5. Genomic information on non-O1/non-O139 *Vibrio cholerae* strains used as references in this study

Strain	Year	Country	Source	MLST	Genome Size (bp)	Completeness	Contamination	BioSample no.
Santiago-089	2018	Chile	Human	ST721	4,071,762	99.45	1.38	SAMN11318966
HC-1A2	2010	Haichi	Human	ST740	3,965,428	99.78	0.32	SAMN00249592
M20227	2019	Australia	Human	N/A	3,948,429	99.32	1.57	SAMN32378343
VcN1	2017	Bangladesh	Environment	ST1438	4,145,933	99.39	1.31	SAMN07786812
HC-43B1	2010	Haichi	Human	ST736	3,931,831	99.58	0.23	SAMN00619210
HE-45	2010	Haichi	Environment	ST730	4,125,202	99.62	0.75	SAMN00621717
M121990	2012	Australia	Human	N/A	4,108,419	99.71	2.77	SAMN32378289
1157-74	1974	India	Human	ST1010	4,012,216	99.34	1.46	SAMN02693889
1311-69	1969	India	Human	N/A	3,973,555	99.45	0.51	SAMN02693887
M152362	2015	Australia	Human	N/A	4,076,578	99.40	1.96	SAMN32378331
M16196	2016	Australia	Human	N/A	4,015,192	99.39	3.40	SAMN32378336
M99474	1999	Australia	Human	N/A	4,012,839	99.54	1.89	SAMN32378292
M056274	1999	Australia	Human	N/A	4,269,669	99.40	3.21	SAMN32378292
M011053	2001	Australia	Human	N/A	4,075,982	99.18	2.94	SAMN32378295
TMA21	1982	Brazil	Environment	ST79	4,023,772	99.15	0.86	SAMN02393808
M06110	2006	Australia	Human	N/A	4,123,505	99.58	2.44	SAMN32378304
AM-19226	2001	India	Human	ST29	4,037,687	99.69	1.11	SAMN02435859
M031916	2003	Australia	Human	N/A	4,055,808	99.69	1.94	SAMN32378299
M16108	2016	Australia	Human	N/A	4,324,626	99.69	7.37	SAMN32378334
CP1110	2010	USA	Human	ST754	3,925,419	99.44	0.87	SAMN02469662
CP1117	2010	USA	Human	ST754	3,925,548	99.42	0.87	SAMN02469665
M041243	2004	Australia	Human	ST770	3,975,152	99.69	3.08	SAMN32378301
M136462	2013	Australia	Human	ST711	3,976,237	99.78	3.11	SAMN32378327
M01655	2001	Australia	Human	ST8	4,008,427	99.54	0.98	SAMN32378294
M182863	2018	Australia	Human	ST777	4,143,246	99.69	3.59	SAMN32378339
M09657	2009	Australia	Human	N/A	4,070,077	99.63	1.04	SAMN32378311
M055620	2005	Australia	Human	N/A	4,100,095	99.63	1.41	SAMN32378302
M12761	2012	Australia	Human	N/A	4,056,274	99.63	1.14	SAMN32378325
M19206	2019	Australia	Human	N/A	4,091,273	99.63	1.32	SAMN32378342
M175282	2017	Australia	Human	N/A	4,063,666	99.59	1.83	SAMN32378338
M041117	2004	Australia	Human	ST1077	3,839,380	99.40	1.18	SAMN32378300
M12590	2012	Australia	Human	N/A	4,171,165	99.69	3.03	SAMN32378324
M138351	2013	Australia	Human	N/A	4,143,714	99.69	2.74	SAMN32378328
M183306	2018	Australia	Human	ST796	4,166,055	99.40	3.26	SAMN32378340
8-76	1976	India	Human	ST5	4,074,796	99.63	1.18	SAMN02693891
M083375	2008	Australia	Human	N/A	4,047,656	99.42	2.35	SAMN32378310
MZO-3	2001	Bangladesh	Human	ST65	4,119,642	99.36	0.90	SAMN02435869
2012EL-1759	2012	Haichi	Environment	ST729	3,982,871	99.71	0.34	SAMN02195196
M147540	2014	Australia	Human	ST841	4,133,020	99.71	4.61	SAMN32378330
N16961	1975	Bangladesh	Human	ST69	4,047,835	99.69	0.24	SAMN02603969
981-75	1975	India	Human	ST74	4,034,493	99.65	0.30	SAMN02693890
VN-00534	2017	Germany	Human	ST590	3,988,274	98.75	1.36	SAMN08622972
VN-00168	2010	Germany	Human	ST496	3,941,253	99.13	2.02	SAMN06484415
VN-00297	1995	Germany	Human	ST490	3,994,678	99.11	1.94	SAMN06484417
VN-00307	2012	Germany	Human	ST490	4,004,351	99.13	1.90	SAMN06484213
VN-00533	2016	Germany	Human	ST589	4,117,309	99.26	1.55	SAMN06484414
VN-00298	1995	Germany	Human	ST491	3,849,167	97.12	1.40	SAMN06484418
VcCHNf9	2013	China	Environment	N/A	4,009,807	98.73	1.38	SAMN09945392
M162017	2016	Australia	Human	N/A	4,185,137	98.75	3.95	SAMN32378335
M066673	2006	Australia	Human	N/A	4,296,634	98.73	2.56	SAMN32378305
M072271	2007	Australia	Human	ST1215	4,050,458	98.39	2.44	SAMN32378307
60555434	2017	Australia	Human	ST618	3,918,371	98.90	1.53	SAMN09404502
S12	2009	Australia	Environment	ST764	4,058,287	98.63	2.03	SAMN05361712
VN-00300	1999	Germany	Human	ST448	3,986,607	99.10	1.85	SAMN06484420
M091373	2009	Australia	Human	N/A	4,129,943	98.80	4.74	SAMN32378312
M192962	2019	Australia	Human	N/A	4,007,565	98.89	3.08	SAMN32378341
PS15	NA	USA	Environment	ST1388	3,910,387	98.21	2.06	SAMN02470610
RC385	1962	USA	Environment	N/A	4,120,133	97.31	2.88	SAMN02435834
M144786	2014	Australia	Human	ST601	4,161,936	98.83	4.00	SAMN32378329
M103422	2010	Australia	Human	ST1340	4,118,719	99.45	1.70	SAMN32378314
M01563	2001	Australia	Human	ST338	3,980,841	99.43	1.86	SAMN32378293
M027753	2003	Australia	Human	ST338	4,078,660	99.43	2.00	SAMN32378297
M03309	2003	Australia	Human	ST338	4,082,108	99.43	2.59	SAMN32378298
