# Supplementary material for: Genomic insights into clinical non-O1/non-O139 Vibrio cholerae isolates in Japan
Source: Microbiol Spectr. 2025 Jun 24;13(8):e00175-25. doi: 10.1128/spectrum.00175-25 (PMC12323619; doi:10.1128/spectrum.00175-25)
Supplement: Supplemental material — Fig. S1 legend. [file spectrum.00175-25-s0004.docx]

**Supplementary Figure 1** **Diagram of VopM, including the repeating Rep1 unit, and amino acid sequences of the VopM region detailing the Rep1 unit.**

Based on the results of the type III secretion system (T3SS) comparison (Figure 2), sequencing analysis was performed for VopM, which was suspected to have the Rep1 unit. The similarity of the VopM Rep1 unit in this study was 17.1–100.0% compared to VopM of AM-19226, the *V. cholerae* reference strain harboring T3SS; NGY2020-029 had seven repeats of the Rep1 unit, while NGY2020-031 had three repeats and NGY2020-056 had two repeats.
