# Supplementary material for: Genomic insights into clinical non-O1/non-O139 Vibrio cholerae isolates in Japan
Source: Microbiol Spectr. 2025 Jun 24;13(8):e00175-25. doi: 10.1128/spectrum.00175-25 (PMC12323619; doi:10.1128/spectrum.00175-25)
Supplement: Supplemental material 2 — Fig. S2 legend. [file spectrum.00175-25-s0005.docx]

**Supplementary Figure 2 Non-O1/non-O139 *Vibrio cholerae* isolated in this study possess various types of type VI secretion system (T6SS).**

Genomic information of the isolates was used to screen for the T6SS. The effector and immune proteins of the T6SS in this study were compared to previous T6SS sequences of large clusters and each auxiliary cluster (AUX) for typing. NGY2020-029 has a type C effector protein of AUX-1, a type E effector protein of AUX-2, and a type C effector protein of Large Cluster (CEC). VgrG-1 of AUX-1 of NGY2020-029 included an actin cross-linking domain. NGY2020-0031 had the type C effector protein AUX-1, type A effector protein AUX-2, and type C effector protein of Large Cluster (CAC), and additionally, AUX-4 was detected. NGY2020-056 had type C effectors of AUX-1, type D effector proteins of AUX-2, type C effector, and type E effector proteins of Large Cluster (CDC), and type C AUX-5 was detected. In the Large Cluster NGY2020-056, multiple immune proteins such as type C, type E, and type A were identified.
